# Primary retroperitoneal mucinous cystadenocarcinoma: report of two cases

**DOI:** 10.1186/1477-7819-5-5

**Published:** 2007-01-15

**Authors:** David Cantú de León, Delia Pérez-Montiel, José Chanona-Vilchis, Alfonso Dueñas-González, Verónica Villavicencio-Valencia, Gladys Zavala-Casas

**Affiliations:** 1 Department of Gynecologic Oncology, Instituto Nacional de Cancerología de México (INCan), Mexico City, Mexico; 2 Department of Pathology, Instituto Nacional de Cancerología de México (INCan), Mexico City, Mexico; 3 Department of Clinical Research, Instituto Nacional de Cancerología de México (INCan), Mexico City, Mexico; 4 Department of Surgical Oncology, Instituto Nacional de Cancerología de México (INCan), Mexico City, Mexico; 5 Department of Gynecology, Centro Médico de la Mujer, Monterrey, Nuevo León, Mexico

## Abstract

**Background:**

Retroperitoneal cystadenocarcinomas are rare lesions, the majority of cases presented as one-patient reports.

**Methods:**

We present two cases of retroperitoneal cystadenocarcinoma, both in women of reproductive age: one with aggressive behavior, and the remaining case, with a more indolent clinical evolution.

**Results:**

One case presented as pelvic tumor, was treated with surgical resection of the disease, but manifested with recurrent disease a few months later despite use of chemotherapy. The second case involved a patient with diagnosis of abdominal tumor; during laparotomy, a retroperitoneal tumor was found and was totally removed. At follow-up, the patient is disease-free with no other treatment.

**Conclusion:**

The behavior and treatment of retroperitoneal cystadenocarcinoma are controversial. We suggest aggressive surgery including radical hysterectomy and bilateral salpingoopherectomy with adjuvant chemotherapy in these cases.

## Background

Retroperitoneal primary cystadenocarcinomas are extremely rare entities [1]. At present, no more than 35 cases of primary retroperitoneal mucinous cystadenocarcinomas have been reported in the English literature [2]. All are females, but two cases have been reported in men [3,4].

In the majority of instances, preoperative diagnosis of these lesions is not possible because computed tomography (CT) scans or magnetic resonance imaging (MRI) is not able to distinguish the exact origin of the lesion. The most common presumptive diagnosis at surgery is abdominal mass.

Due to its rarity, optimal treatment options, survival, and exact prognosis continue to be uncertain. We present two cases of primary retroperitoneal mucinous cystadenocarcinomas treated at a single institution; these had different clinical presentations and totally different outcomes with the most adequate treatment for each case according to clinical presentation. To the best of our knowledge, these are the first of such cases reported in Latin-American women.

## Case presentation

### Case one

A 36-year-old Hispanic woman had an appendicitis-associated appendectomy at 6 years of age, and post-Cesarean section uterine atony-related total abdominal hysterectomy at age 26 years. Other medical, surgical, or family histories were unremarkable.

**Figure 1:**
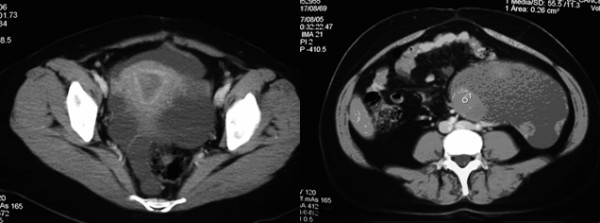
Computer tomography (CT) scan of pelvis. a). showing normal uterus and a pelvic mass located in the area of the left adnexa. b). showing cystic mass with solid areas.

The patient was seen with a 6-month history of abdominal distention and discomfort. At physical examination, no abdominal masses or ascites were detected. A pelvic ultrasound (US) was performed, and an adnexal cystic mass of 19 × 16 × 12 cm with solid component was observed in the area of the left adnexa. There was no ascites or any other abnormalities observed in the pelvic cavity. Serum CA-125 was 4.13 U/ml, and routine preoperative laboratory tests were obtained and reported as normal, as was the chest x-ray. A CT scan was conducted as part of the preoperative evaluation; the scan confirmed the US findings and revealed solid areas in a cystic tumor (Figures 1a and 1b).

An exploratory laparotomy was performed with the diagnosis of a suspicious malignant adenexal mass. During the procedure, a tumor measuring 19 × 16 × 12 cm was found in the left retroperitoneal space, the tumor completely separated from the ovaries or other intrapelvic structures. Complete and intact removal of the lesion was obtained; this was sent for frozen section, and was subsequently reported as mucinous cystadenocarcinoma. At the time of the surgery, both ovaries and fallopian tubes were grossly normal, as were all intra-abdominal structures. The patient's postoperative course showed no complications or adverse events. Six courses of adjuvant chemotherapy were administered with carboplatinum at a dose of 6 area under the curve (AUC) and paclitaxel at 175 mgs/m^2^. CA-125 was obtained after the fifth chemotherapy course and was reported as 5.3 U/ml. A pelvic US was performed 8 months later, and a new pelvic mass measuring 7 × 6 × 6 cm at the same site was found. An exploratory laparotomy was conducted and a sigmoid resection was required due to a mass involving this. Infracolic omentectomy was also carried out due to multiple nodules. The surgery was considered optimal cytoreduction with no visible lesion remaining. The Pathology report stated mucinous cystadenocarcinoma.

Second-line chemotherapy was initiated with oral etoposide (50 mg daily for 21 days). After one cycle, disease progression was identified and the patient decided to stop chemotherapy. Tamoxifen (20 mg/day) was instituted as palliative management.

### Pathological findings

**Figure 2:**
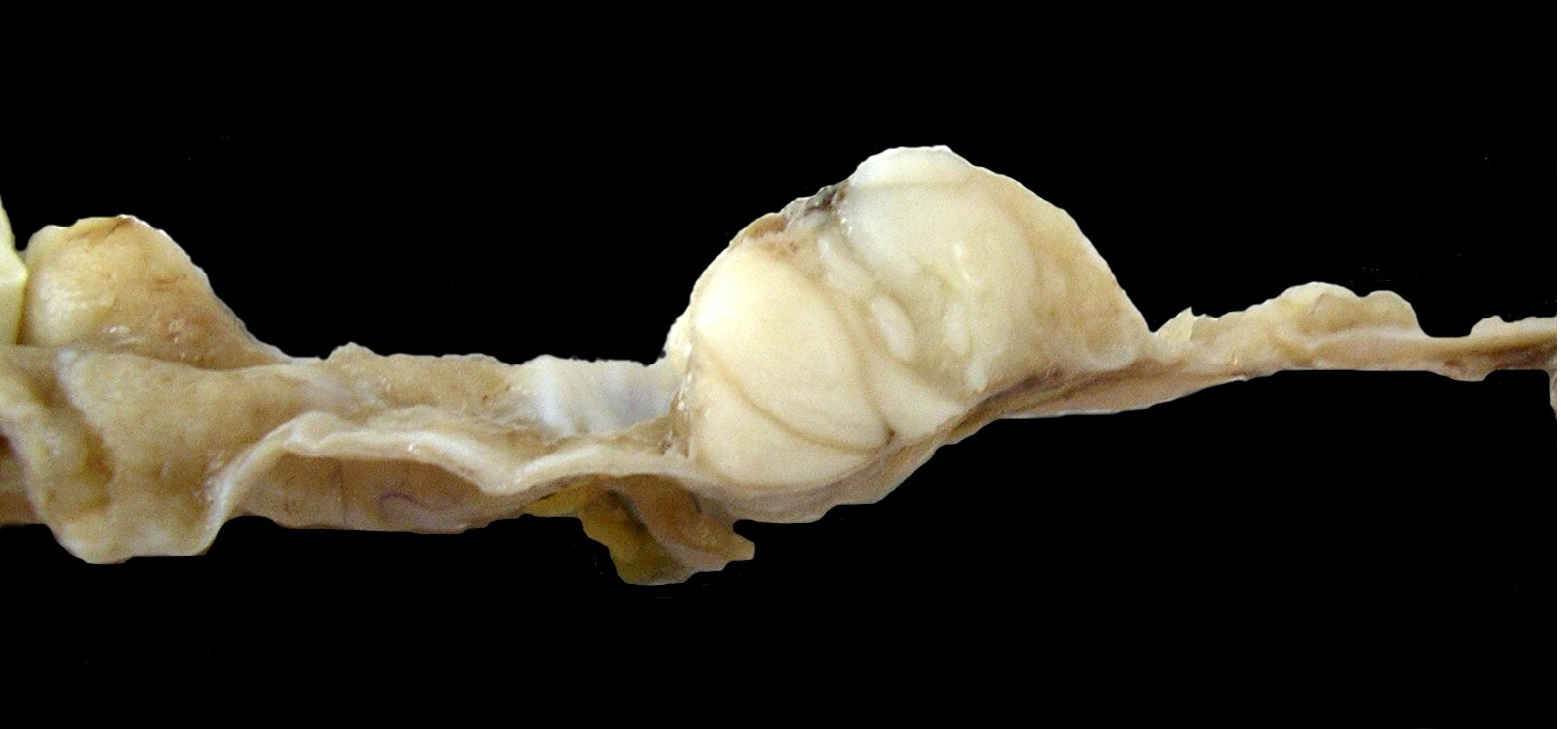
Gross section of the cystic lesion shows nodular area.

**Figure 3:**
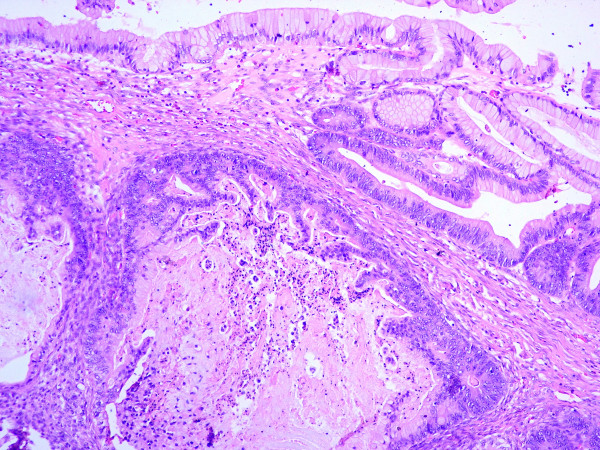
Microscopically, the tumor shows transition between benign and malignant areas of the mucinous tumor (Hematoxilin and eosin stain 20×).

On gross examination, an ovoid, well-defined tumor with measurements of 19 × 16 × 12 cm with smooth grey surface was received. Internal surface comprised a multilocular mass with thin walls and mucinous material only, while a small area exhibited solid nodules in the wall (Figure 2). Microscopically, tumor walls were covered with a single line of mucinous cells with small basal nuclei and mucinous cytoplasm. In addition, ovarian-like stroma was identified in the wall. These epithelial areas showed transitions with intraepithelial carcinoma (Figure 3) and areas of borderline mucinous tumor; high-grade adenocarcinoma with dedifferentiation in desmoplastic stroma was identified. High-grade adenocarcinomatous component infiltrated the tumor capsule was seen.

The recurrent tumor's surgical specimen comprised a multiorgan pelvic resection with an ill-defined white mass with gross infiltration to muscle and fatty tissue of the pelvis wall, left ovary, fallopian tube, omentum, and in wall of the colon, without lesion in the mucosa. Microscopically, high-grade adenocarcinoma similar to high-grade areas of the previous lesion was identified, the ovary demonstrating direct infiltration from the abdominal mass.

### Case two

**Figure 4:**
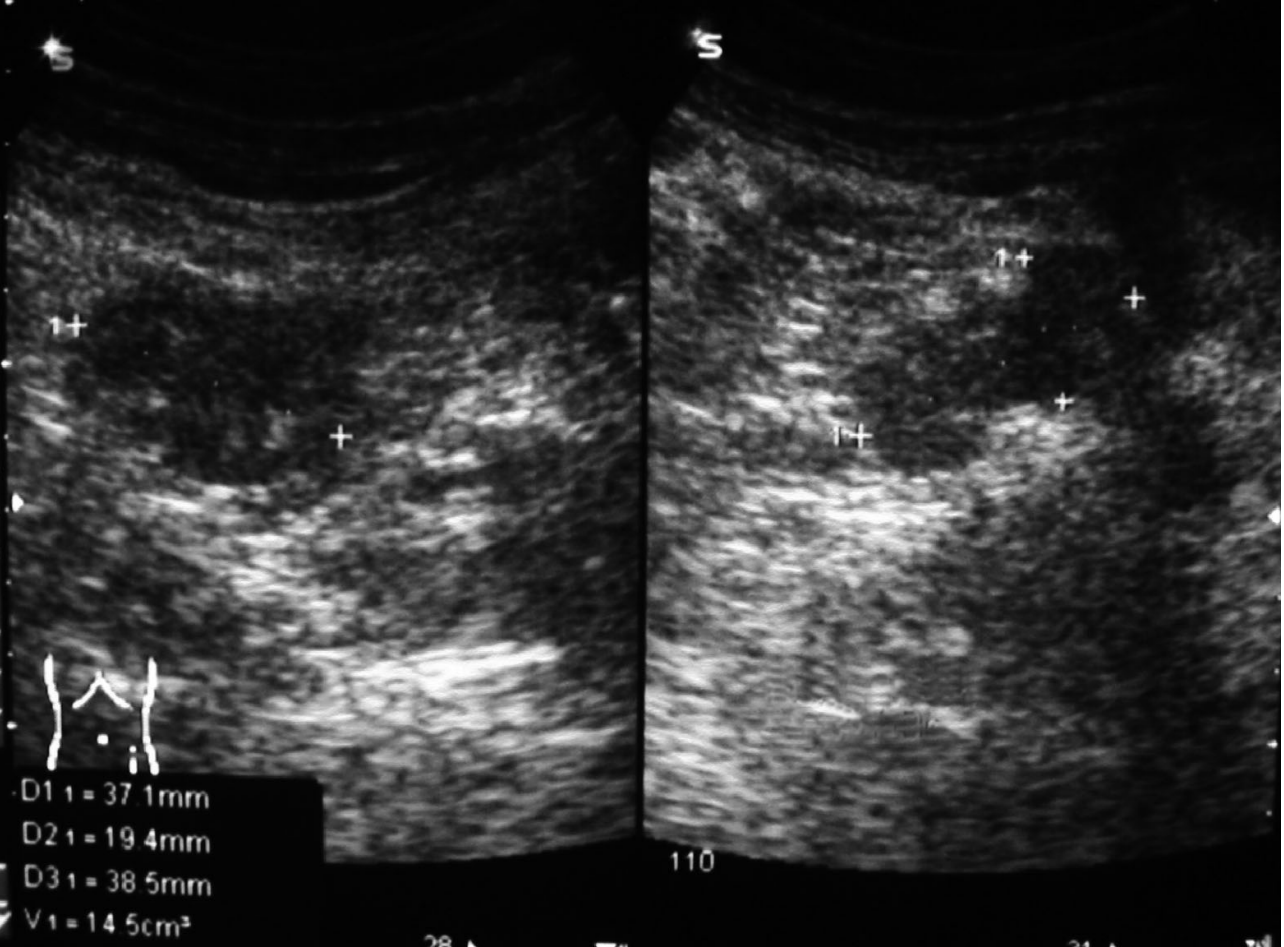
Ultrasound (US) reveals a cystic mass with solid areas in the second case.

The patient, a 21-year-old female with no remarkable previous medical or surgical history complained of diffuse abdominal discomfort 1 month prior to the patient's presentation at the Emergency Service due to acute abdominal pain and intestinal occlusion-compatible clinical data. Abdominal and pelvic US was performed, revealing a cystic mass with solid areas (Figure 4). An emergency laparotomy was performed; during the surgery, a retroperitoneal tumor measuring 26 × 18 × 16 cm was observed. The latter was totally removed, ascitic fluid was detected and drained, and cytology was reported as negative for malignant cells. The tumor was located near the pancreas, but was not attached to this organ or to any other intra-abdominal or pelvic structures. Ovaries, uterus, colon, and appendix were macroscopically normal, as were other intra-abdominal structures. Postoperative serum CA-125 was 105 U/ml (0–21), while CA-15-3 was 32.2 U/ml (0–53) and CA-19-9 was 5.4 U/ml (0–37).

Systemic adjuvant chemotherapy was proposed; nonetheless, the patient refused this treatment. Therefore, close follow-up was advised, and the patient has been seen at the Medical Oncology Service over the last 6 months with serial CA-125 serum levels at latest measurement of 13.3 U/ml. An abdominal CT was performed, and no evidence of disease was found.

### Pathological findings

Grossly, the tumor was a well-defined multilobular mass of 26 × 18 cm. Cut surface exhibited a multicystic tumor with thin walls and solid white areas. Microscopically, the lesion was a mucinous tumor with well-differentiated mucous glands with cribiform or papillary architecture, and cells had pseudo-stratified, large regular nuclei with mucinous cytoplasm, these areas demonstrating transition to cystic areas lined by a single line of mucinous cells without atypia. No capsular invasion was identified.

## Discussion

Retroperitoneal tumors of epithelial origin are rare, because no epithelial cells are found in this area; nevertheless, cases of lesions with Müllerian-type epithelium have been reported, Roth *et al*., the first group in 1977[5]. The exact origin of these tumors remains unclear [1], and many theories have been postulated. One of the most accepted of these is that of ectopic or supernumerary ovarian tissue [1], because these tumors resemble ovarian tumors mainly due to the presence of ovarian-like stroma. Notwithstanding this, it is noteworthy that normal ovarian tissue or remnants of normal ovarian tissue are not found in any reported cases, including ours [1,4]. Another issue against theory consists of the fact that at least two cases in males have been reported previously [3,4]. Another theory is celomic metaplasia, in which some clusters of celomic epithelial cells are deposited in the retroperitoneal area, developing an inclusion cyst. These cells eventually experience metaplastic changes resulting in mucinous tumors that finally have cytologic changes, thus gaining the malignant phenotype [4,6]. Other authors have proposed that the peritoneal epithelium possesses the potential for Müllerian differentiation, as do all epithelial ovarian tumors [7]. This concept has been supported by some authors based on immunohistochemical and electron-microscopy evaluation of the tumors [4]. Another is the possibility of mucinous tissue overgrowing other components of a teratoma [4].

Age-at-presentation ranges from teenaged to elderly patients, and the most common complaint at presentation has been abdominal discomfort and a slow-growing pelvic or abdominal mass [4,6]. In one of our cases, presentation was acute abdominal pain and intestinal occlusion. Matsubara in his review mentions that no cases examined involved severe pain; [6] therefore, we might say that this is the first case reported to date with this clinical presentation.

Preoperative diagnosis is rarely suspected because of the non-specific symptoms and the scarce aid of imaging examinations. Although US, CT scan, and MRI clearly detect cystic masses in ovarian or pelvic organs, diagnosis of retroperitoneal tumor is extremely difficult. This was mentioned by Matsubara *et al*., in that the authors were solely able to find two reports in the literature with suspicious preoperative retroperitoneal tumor in their review of 16 cases from 1966 to the report in 2005 [6]. Others authors such as Thamboo mentioned that diagnosis of retroperitoneal cyst was suspected by means of CT scan [3]. Although no specific data have been reported regarding the characteristics of lesions that may suggest a retroperitoneal tumor, displacement of ureter, kidney, great vessels, or colon may be of some aid in the preoperative diagnosis [6].

Tumor markers are not very helpful in differentiating the exact origin of the lesion, because CA-125, CA19-9 may or may not be elevated and may or may not have increased values in other neoplasms such as epithelial ovarian tumor (serous or mucinous). On the other hand, tumor markers help in detecting a recurrent tumor, as in ovarian cancer or colon cancer [4].

It is clear that surgical treatment of these tumors is the cornerstone, but the question remains: how extensive must the surgery be? Everyone agrees on total removal of the lesion, but some authors such as Gotoh *et al*., [7] recommend oophorectomy to improve survival. Lee *et al*., [8] recommend not only oophorectomy, but also total hysterectomy. On the other end of the surgical-treatment spectrum, Kessler *et al*., [9] recommend that if uterus and ovaries are macroscopically normal and because follow-up in the majority of reported cases is deficient and the serious consequences of this surgical procedure, hysterectomy and salpingo-oophorectomy cannot be justified for treatment of primary retroperitoneal mucinous cystadenocarcinomas [9]. The only reason for this procedure to be performed is in postmenopausal women or patients who have completed child-bearing. We can also find reports presenting more conservative surgery, such as that proposed by Law *et al*., who advocate for laparoscopic excision of the tumor and complete evaluation of abdominal and pelvic organs, sparing fertility in these women [10].

Adjuvant chemotherapy is not a standard treatment and is beneficial solely in cases in which the tumor has been ruptured during surgery [9], or when invasion to adjacent structures is evident. Another point in favor of adjuvant treatment is evidence that primary retroperitoneal mucinous cystadenocarcinomas and ovarian mucinous tumor have similar mechanisms in their histogenesis; this was reported by Tenti *et al*. [11], who had found K-ras oncogene mutation at codon 12 and demonstrated immunoreactivity for intestinal cell markers. In one of our cases, in which the tumor was completely excised without rupture but with capsular invasion, chemotherapy was given with no benefit because the patient experienced recurrence a few months after completion of adjuvancy. We also had the remaining case with no complementary treatment; the patient is at present disease-free. Thus, the questions remain: In which patients may recurrence occur? Which cases must have addition of cytotoxic chemotherapy? Or shall we just have close follow-up of these patients, as proposed by some authors [4,12]?

The answers of these questions must wait for the moment until we can determine whether the patient is at risk for recurrence or not, and further study is needed to establish optimal-treatment protocols on these rare neoplasms. We are in favor of aggressive adjuvant chemotherapy with hysterectomy and bilateral oophorectomy only when macroscopic involvement of these structures is found during the procedure.

## Conflict of interests

The author(s) declare that they have no competing interests.

## Authors' contributions

**DCL **– Contributed to manuscript conception, writing, organizing, drafting and revision of the manuscript.

**DPM **– Reporting pathologist, involved in discussion leading to manuscript preparation and revision.

**JCHV **– Reporting pathologist, contribution on images of both cases, critical review of manuscript.

**ADG **– Acquisition of consent, patient clinical managed and critical revision of manuscript.

**VVV **– Follow up of patients, collection of data and revision of the manuscript.

**GZC **– Follow up, collection of data and revision of manuscript.

All authors have read and approved the final manuscript.
